# A narrative review of evidence to support increased domestic resource mobilization for family planning in Nigeria

**DOI:** 10.1186/s12905-023-02396-y

**Published:** 2023-05-06

**Authors:** Chinyere Ojiugo Mbachu, Ifunanya Clara Agu, Chinazom N. Ekwueme, Anne Ndu, Obinna Onwujekwe

**Affiliations:** 1 Health Policy Research Group, University of Nigeria, Enugu, Nigeria; 2 Department of Community Medicine, University of Nigeria, Enugu Campus, Enugu, Nigeria; 3 Department of Health Administration and Management, University of Nigeria Enugu Campus, Enugu, Nigeria

**Keywords:** Family planning funding, Unmet need for contraceptives, Contraceptive prevalence, Nigeria

## Abstract

**Background:**

Family planning (FP) is an important public health intervention that is proven to reduce unplanned pregnancies, unsafe abortions, and maternal mortality. Increasing investments in FP would ensure stability and better maternal health outcomes in Nigeria. However, evidence is needed to make a case for more domestic investment in family planning in Nigeria. We undertook a literature review to highlight the unmet needs for family planning and the situation of its funding landscape in Nigeria. A total of 30 documents were reviewed, including research papers, reports of national surveys, programme reports, and academic/research blogs. The search for documents was performed on Google Scholar and organizational websites using predetermined keywords. Data were objectively extracted using a uniform template. Descriptive analysis was performed for quantitative data, and qualitative data were summarized using narratives. Frequencies, proportions, line graphs and illustrative chart were used to present the quantitative data.

Although total fertility rate declined over time from 6.0 children per woman in 1990 to 5.3 in 2018, the gap between wanted fertility and actual fertility increased from 0.2 in 1990 to 0.5 in 2018. This is because wanted fertility rate decreased from 5.8 children per woman in 1990 to 4.8 per woman in 2018. Similarly, modern contraceptive prevalence rate (mCPR) decreased by 0.6% from 2013 to 2018, and unmet need for family planning increased by 2.5% in the same period. Funding for family planning services in Nigeria comes from both external and internal sources in the form of cash or commodities. The nature of external assistance for family planning services depends on the preferences of funders, although there are some similarities across funders. Irrespective of the type of funder and the length of funding, donations/funds are renewed on annual basis. Procurement of commodities receives most attention for funding whereas, commodities distribution which is critical for service delivery receives poor attention.

**Conclusion:**

Nigeria has made slow progress in achieving its family planning targets. The heavy reliance on external donors makes funding for family planning services to be unpredictable and imbalanced. Hence, the need for more domestic resource mobilization through government funding.

## Background

More than 200 million women in developing countries want to avoid or delay pregnancy. Yet, they lack access to effective and safe family planning services [1]. In Nigeria, fewer than two out of every ten married women use modern contraceptives, and 19% of women have an unmet need for family planning [2]. The reasons for this include supply-side issues such as unavailability of FP services and information, and demand-side issues such as lack of funds and poor support from partners or communities [2]. Limited access to FP services prevents women of reproductive age from delaying pregnancy, limiting family size and safe spacing [3–6].

In order to address the socio-cultural issues that limit access to family planning services, the Federal government designed a strategy for engaging with stakeholders to discuss issues about large family size, religious beliefs, and women's power to make decision about their sexual and reproductive health [7]. This approach has been effective in addressing some of the cultural barriers to contraception and the increase in contraceptive use in some communities in Nigeria has been attributed to the involvement of community leaders to promote family planning [8].

Family planning (FP) is an important public health intervention that is proven to improve maternal and child health outcomes by reducing unplanned pregnancies and unsafe abortions [9–13]. To ensure progressive improvements in maternal and child health outcomes through family planning, there is need for sustained and dedicated funding of family planning programmes [14, 15]. Evidence from the United States international family planning assistance in 2014 showed that investments in family planning services and contraceptive supplies saves millions of lives [16]. Through improving access to contraceptives for 30 million women and couples, 7 million unintended pregnancies, 2 million unsafe induced abortions and 13 thousand maternal deaths were averted [16].

In Nigeria, the national budget for family planning was cut short by 90% in 2019, owing to lack of counterpart funding to match grants from donors [17]. This resulted in the stock-out of contraceptive commodities in the primary health centers, and worsened access to family planning services for women [18]. Moreover, with the withdrawal of donor funds, domestic resource mobilization (DRM) for family planning services and contraceptive supplies became an urgent need for the Reproductive, Maternal, Newborn, Child and Adolescent Health (RMNCAH) program in Nigeria to improve [2]. Granted that increasing investments in FP would ensure stability and better maternal health outcomes in Nigeria, evidence is needed to make a case for more domestic investment in family planning.

Evidence generation is a critical component of the National and State roadmaps for improved domestic resource mobilization for family planning in Nigeria [19], and reliable evidence is needed to motivate policymakers and domestic funders to allocate more resources towards family planning services and contraceptive supplies. Therefore, we undertook a literature review to determine the unmet needs for family planning and analyze the funding landscape in Nigeria, with a view to highlight the need for increased domestic funding of family planning services. The findings will be invaluable to policymakers and family planning program officers in advocating for domestic funding for family planning interventions**.**

We undertook a narrative review of literature from February to May 2022 to generate evidence that showcases the need to allocate more domestic funds to family planning services in Nigeria.

Our review sought to answer two key questions,

1. What is the unmet need for family planning in Nigeria?
2. How is the family planning programme in Nigeria funded, and what does this imply for reliability and predictability of funding?

### Main text

To answer these questions, we analyzed the trends in fertility rate and contraceptive prevalence rate from 1990 to 2018 and estimated the gaps in wanted and actual fertility. Then we undertook a funding landscape analysis using the bespoke framework that highlights the types and characteristics of funding organizations (in terms of reliability and predictability), as well as their interests or areas of funding.

### Document search

Electronic search was performed on Google Scholar, organizational websites and blogs to source for relevant documents, such as peer-reviewed articles, reports from national surveys, and reports from family planning programmes and interventions.

The reports from the Nigeria Demographic and Health Survey (NDHS) were collated from 1990 to 2018, while the reports for the Multiple Indicators Cluster Survey (MICS) were collated for 1999 to 2016. Peer-reviewed journal articles and website articles that were published in English language from January 2008 to June 2021 were included in the review, and the scope of the review was limited to Nigeria only. The search for articles was performed using various combination of key terms including, “family planning”, “contraceptives”, “fertility rate”, “contraceptive prevalence rate”, “financing”, “funding landscape”, “funders”, “unmet need”, “unwanted pregnancy”, “family planning investment”.

A total of 30 documents were reviewed including, 16 journal articles, ten web blogs, and six national survey reports [2, 7, 8, 19–47].

### Data extraction and synthesis

Data were objectively extracted by two independent researchers using a uniform template that was designed in Microsoft Excel.

The template was structured according to themes, including a description of the article under review, and the findings from the review were synthesized according to the thematic areas, namely,

- Fertility rates
- Contraceptive prevalence rates
- Variations in fertility and contraceptive prevalence rates
- Nature of funding, including
  - ◦ Name and type of funding organization
  - ◦ Interest of funding organization (areas/aspects of family planning services that are funded)
  - ◦ Type of funding (e.g., grant, loan)
  - ◦ Duration of funding
  - ◦ Funding route (e.g., third party financing, direct facility financing, etc.)
  - ◦ Conditions of funding (e.g., counterpart funds, results-based)

Narrative summaries are presented for the qualitative data. Proportions are reported for quantitative data.

## Results

### Trends in fertility rates

**Fig. 1:**
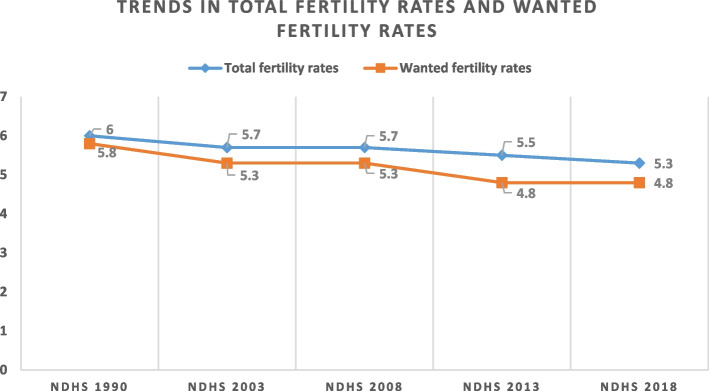
Trends in total fertility rates and wanted fertility rates in Nigeria from 1990 to 2018

Figure 1 shows that the total fertility rate in Nigeria has gradually declined over time from 6.0 children per woman in 1990 to 5.3 in 2018, and that the wanted fertility rate has decreased from 5.8 children per woman in 1990 to 4.8 per woman in 2018 [2, 20–24].

However, the gap between wanted fertility and actual fertility has increased over time from 0.2 in 1990 to 0.5 in 2018. This signifies that a Nigerian woman has 0.5 more children than she wants to have.

### Trends in contraceptive prevalence rate and unmet need for family planning

**Fig. 2:**
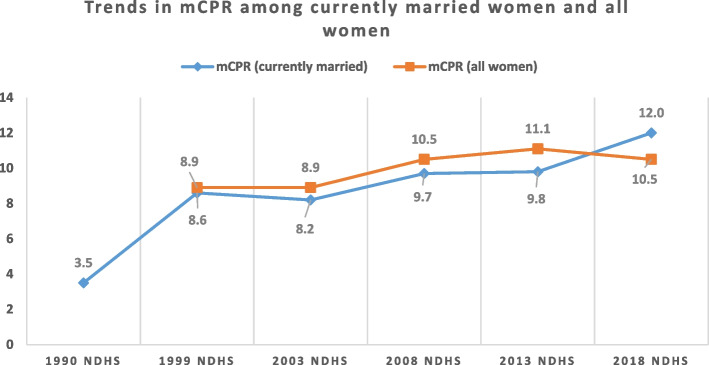
Trends in modern contraceptive prevalence rate among currently married women and all women in Nigeria from 1990 to 2018

As shown in Fig. 2, modern contraceptive prevalence rate (mCPR) improved from 3.5% in 1990 to 12.0% in 2018, which indicates an increase of 8.5% in 28 years [2, 20–24].

This translates to a yearly increase of 0.3%, which if sustained will not result in the achievement of the country’s mCPR target of 27% by 2024 [19].

Even though mCPR is traditionally reported for currently married women, it is noteworthy that mCPR among all women decreased from 11.1% in 2013 to 10.5% in 2018.

**Fig. 3:**
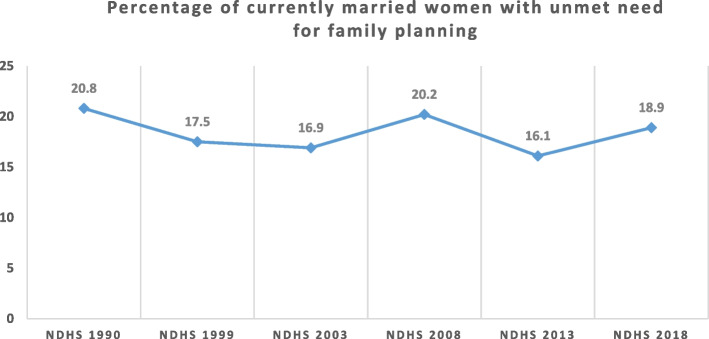
Trend in percentage of currently married women with unmet need for family planning from 1990 to 2018

Figure 3 shows that the unmet need for family planning among currently married women reduced by 3.9% in 2003 and increased by 3.3% in 2008; and has since followed the pattern of falling and rising. Between 2013 and 2018, unmet need for family planning increased by 2.8% [2, 21].

### Geographic variations in fertility and contraceptive prevalence rates

Over time, total fertility rate has been consistently higher in the rural areas compared to the urban areas, whilst mCPR has been higher in urban areas than in rural areas, expectedly.

Wide variations in TFR are also seen across geopolitical zones, with the northern zones having substantially higher rates than the south. It is noteworthy that over the years, the southern zones have consistently had lower fertility rates than the national average. Women in the north-east and north-west geopolitical zones reported having an average of two more children than their counterparts in the south.

Regarding contraceptive prevalence, there are wide regional variations in mCPR across the geopolitical zones and the States. The northern zones have consistently reported lower mCPR than the south, and the north-east and north-west geopolitical zones have had the lowest mCPR in the north.

These geographic disparities in fertility rates and mCPR are summarized in Tables 1 and 2, respectively.

**Table 1:** Geographic distribution of total and wanted fertility rates from 1990 to 2018 in Nigeria

**Geographic area**	**Total fertility rate**						**Wanted fertility rate**					
	**1990**	**1999**	**2003**	**2008**	**2013**	**2018**	**1990**	**1999**	**2003**	**2008**	**2013**	**2018**
Urban	5.0	4.5	4.9	4.7	4.7	4.0	4.8	4.2	4.6	4.4	4.1	4.0
Rural	6.3	5.4	6.1	6.3	6.2	5.4	6.1	5.1	5.7	5.8	5.3	5.4
**Geopolitical zones**												
North-central	^a^	4.5	5.7	5.4	5.3	4.7	^a^	4.2	5.2	5.1	4.2	4.7
North-east	6.5	6.8	7.0	7.2	6.3	5.6	6.2	6.4	6.7	6.7	4.6	5.6
North-west	6.6	6.5	6.7	7.3	6.7	5.9	6.6	6.0	6.6	6.8	6.3	5.9
South-east	5.6	4.6	4.1	4.8	4.7	4.3	5.2	4.2	3.5	4.5	4.3	4.3
South-south	^a^	^a^	4.6	4.7	4.3	3.6	^a^	^a^	3.9	4.3	3.6	3.6
South-west	5.5	4.5	4.1	4.5	4.6	3.5	5.2	4.2	3.9	4.2	4.0	3.5
Total	6.0	5.2	5.7	5.7	5.5	5.3	5.8	4.8	5.3	5.3	4.8	4.8

^a^Not available as the six geopolitical zones came into existence after 1999

**Table 2:** Geographic distribution of mCPR among married women from 1990 to 2018 in Nigeria

**Geographic area**	**mCPR among married women**					
	**1990**	**1999**	**2003**	**2008**	**2013**	**2018**
Urban	9.6	15.7	13.9	16.7	16.9	18.2
Rural	1.9	5.6	5.7	6.5	5.7	7.8
**Geopolitical zones**						
North-central	^a^	10.9	10.3	10.5	12.4	13.8
North-east	1.3	2.2	3.0	3.5	2.7	7.8
North-west	0.7	2.5	3.3	2.5	3.6	6.2
South-east	3.9	9.1	13.0	11.8	11.0	12.9
South-south	^a^	^a^	13.8	15.5	16.4	15.8
South-west	10.5	15.5	23.1	21.0	24.9	24.3
Total	3.5	8.6	8.2	9.7	9.8	12.0

^a^Not available as the six geopolitical zones came into existence from 1999

### Funding landscape for family planning in Nigeria

#### Federal Government Budgetary Allocation to Family Planning (2015 to 2020)

Family planning was not an item in budgets before 2015. Allocation by federal government to FP was on the increase from 2015 when line listing for family planning commenced in the national budget. However, there was a sharp decline in 2019 due to the removal of counterpart funding to match grants from international donor agencies which was budgeted for in 2018. See Table 3 below.

**Table 3:** Budgetary allocation to family planning (2015 – 2020)

Year	Budget Line Item	Amount (N)
2015	MDG-IMNCH: ON-GOING PROCUREMENT AND DISTRIBUTION OF CONTRACEPTIVE COMMODITIES; CAPACITY BUILDING FOR SERVICE PROVIDERS AND INFORMATION MANAGEMENT	624,739,731
		**624,739,731**
2016	COUNTERPART FUND FOR THE PROCUREMENT AND NATIONAL DISTRIBUTION OF CONTRACEPTIVE COMMODITIES BASED ON 2016 FORECAST	791,000,000
	DEVELOPMENT OF COSTED IMPLEMENTATION PLAN FOR NIGERIA FP BLUEPRINT	1,217,662
	LAST MILE DISTRIBUTION OF CONTRACEPTIVE COMMODITIES	1,534,799
	TRAINING OF FAMILY PLANNING (FP) SERVICE PROVIDERS	1,827,224
	TRAINING OF COMMUNITY HEALTH EXTENSION WORKERS (CHEWS) ON LONG-ACTING REVERSIBLE CONTRACEPTIVES (LARC)	1,827,224
		**797,406,909**
2017	SUPPORT TO STATES IN THE DEVELOPMENT OF A COSTED IMPLEMENTATION PLAN FOR THE NIGERIA FAMILY PLANNING BLUEPRINT	9,000,000
	REPRODUCTIVE HEALTH- TRAINING OF COMMUNITY HEALTH EXTENSION WORKERS ON FAMILY PLANNING METHODS IN 6 ZONES	30,000,000
	10-DAY TRAINING OF COMMUNITY HEALTH EXTENSION WORKERS ON THE PROVISION OF LONG ACTING REVERSIBLE CONTRACEPTIVES (LARCS)	5,000,000
	TRAINING OF COMMUNITY HEALTH EXTENSION WORKERS (CHEWS) ON LONG-ACTING REVERSIBLE CONTRACEPTIVES (LARCS)	1,827,224
	LAST MILE DISTRIBUTION OF CONTRACEPTIVE COMMODITIES	5,296,827
	CO-FUNDING FOR THE PROCUREMENT & NATIONAL DISTRIBUTION OF CONTRACEPTIVE COMMODITIES BASED ON 2016 FORECAST	915,000,000
		**966,124,051**
2018	IMPROVE FAMILY PLANNING SERVICES THROUGH CONTRACEPTIVES USE INTERVENTIONS & COUNTERPART FUNDING	500,000,000
	COUNTERPART FUNDING TO MATCH GRANTS FROM UNFPA, USAID, BMGF & UNICEF	2,400,000,000
		**2,900,000,000**
2019	IMPROVE FAMILY PLANNING SERVICES THROUGH CONTRACEPTIVES USE INTERVENTIONS & COUNTERPART FUNDING	300,000,000
		**300,000,000**
2020	PROCUREMENT AND DISTRIBUTION OF FAMILY PLANNING COMMODITIES THROUGH COUNTERPART FUNDING TO UNFP	1,200,000,000
		**1,200,000,000**

#### Typologies of funding organization

Funding for family planning services in Nigeria comes from both external and internal sources in the form of cash (grants or loans) or commodities. Internal sources of funding include the Federal and State governments, while external sources include multilateral and bilateral international organizations, and international Non-Governmental Agencies.

**Fig. 4:**
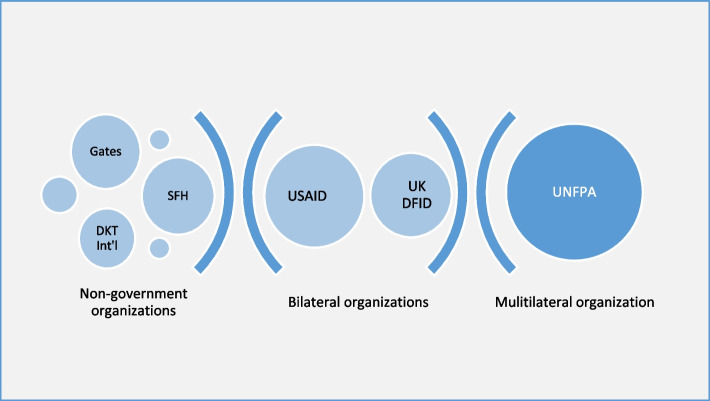
External sources of funding for family planning in Nigeria

The key international and donor agencies that fund family planning services in Nigeria are shown in Fig. 4, and the size of the circle indicates the relative contribution of the funder to family planning services in Nigeria.

UNFPA is the major funder of family planning services in Nigeria, followed by the USAID. UNFPA provides family planning assistance in 19 States plus the Federal capital territory (FCT), while USAID partners with a variety of non-governmental and community-based organizations across the 36 States and the FCT [31].

#### Nature of funding (type, duration, routes and conditions for funding) for family planning

The nature of external assistance for family planning services depends on the preferences of funders, and whether the funding is in form of cash or commodities. External assistance in the form of grants and loans are typically provided to only Federal or State governments, while commodities are provided to public and/or private health facilities through government agencies or implementing partners.

Table 4 shows some similarities and variations among funding agencies in funding for family planning services. With the exclusion of the Saving One Million Lives Programme for Results (SOML PforR), government funding for family planning has been in the form of annual budgetary allocations. Whereas funding from external donors has been in the form of grants. Irrespective of the type of funder and the length of funding, donations/funds are renewed on annual basis.

**Table 4:** Nature of funding for family planning services in Nigeria

Name of funder	Type of funding	Duration of funding	Funding routes	Conditions for funding
Federal government	Budgetary allocation	Annual	State government	Counterpart funding
	Grants to States (SOML PforR)	2016 – 2021 (renewable annually)	State government	Performance-based financing
State government	Budgetary allocation	Annual funding for provision of FP services	Direct facility financing of FP services	Counterpart funding
World Bank (SOML PforR)	Loan	2016 – 2021	Federal government	Loan facility
UNFPA	Grants	Annual procurement over the next 4 years	Supply of FP commodities through State govt	Counterpart funding
USAID	Grants	Annual or biannual procurement	Supply of FP commodities through IPs	Counterpart funding
UK-DFID	Grants	Annual procurement/ commitment	Supply of FP commodities through IPs	Counterpart funding
Gates Foundation	Grants	Annual commitment over the next 5 years (2021–2026)	Funding of FP interventions through IPs	Result-based
SFH	Grants	Annual	Third-party financing	Result-based
DKT International	Grants	Not specified	Direct facility financing for FP services	Result-based
Marie Stopes International (MSI)	Grants	Not specified	Direct supply of FP commodities to organizational clinics and mobile outreach services	Result-based

Various funding routes are employed by external donors, notably the supply of family planning commodities through the State governments or through implementing partners.

Counterpart funding and output-based financing are the two most common conditions for funding.

#### Interests of funding organization (areas/aspects of family planning services that are funded)

The areas or aspects of family planning services that are funded by international organizations and non-government agencies include, (i) procurement of commodities; (ii) distribution and supply chain management (SCM); (iii) training of health workers; (iv) demand creation and community mobilization; (v) other advocacy interventions; and (vi) research. Whereas, government funding is used to procure and distribute commodities, and pay the salaries of health workers.

Table 5 shows that procurement of commodities receives the most attention for funding, while demand creation and research receive the least attention. It is also noteworthy that although State governments are primarily responsible for the distribution of commodities, many States do not allocate or release funds for this purpose. Hence, it can be said that this critical aspect of family planning services is very poorly attended to in Nigeria, and this may well explain the problems of unavailability of commodities at service points.

**Table 5:** Funding agencies and their areas of focus in family planning services in Nigeria

	**Procure-ment**	**Distribution and SCM**	**Trainings**	**Salaries**	Demand creation*	**Advocacy to govt**	**Research**
Federal govt	√	X	X	√	X	NA	X
State govt	X	√	X	√	X	NA	X
UNFPA	√	X	√	X	X	√	√
USAID	√	X	√	X	X	√	√
UK DFID	√	X	X	X	X	X	X
Gates	√	X	X	X	X	X	√
SFH	√	√	√	X	√	X	X
DKT	√	√	√	X	√	X	X
MSI	√	√	√	X	√	X	X

NA - Not applicable

X - the organization is not focused in that area

√ - An organizational area of focus

*Activities that increase clients' desire to use family planning - health education, awareness creation etc

The Nigerian government maps out funds for the procurement and distribution of FP commodities to States. About US$4 million was approved in 2021 for the procurement of family planning commodities [32].

The UNFPA is primarily involved in the procurement of family planning commodities for the public sector. It also provides technical assistance to focus States in the form of training of health workers [26].

The U.S. Agency for International Development (USAID) is a bilateral organization that partners with NGOs to provide FP commodities to both public and private healthcare providers. It also funds programs that seek to improve the quality of FP services and to hold State governments’ accountable to ensuring that FP commodities reach the last mile [25, 26]. The efforts of the USAID-funded Health Policy Plus’ advocacy to Cross-River State government resulted in the allocation of $600,000 for the distribution and security of FP commodities in 2013 and 2014 [25].

The (UK) Department for International Development (DFID) provides the majority of the FP commodities that are supplied to private healthcare providers in Nigeria [27].

Society for Family Health (SFH) and DKT International are social marketing organizations that provide and distribute FP commodities to private facilities [25, 27, 30]***.*** They are also involved in advocacy and training of health workers with primary focus on private providers [26, 30]***.***

Marie Stopes International offers a wide range of sexual and reproductive health services including FP to communities in urban locations, and it has become a major provider of long acting and permanent contraception in health facilities. The organization delivers FP services through static clinics, mobile outreach teams and social franchising [46, 47].

The Gates Foundation focuses on the public sector, and its donations have been used to procure FP commodities [26]. Through the funding that was provided for the Nigeria Urban Reproductive Health Initiative (NURHI), access to FP commodities and services increased in the six intervention cities, resulting in an increase of mCPR by 20% in three years [8, 26, 29].

## Conclusions

Ensuring that every sexually active woman in Nigeria has access to high-quality family planning and contraceptive services is imperative as it save lives and promotes positive maternal health outcomes. Our review highlights that Nigeria’s progress in achieving the targets of family planning has been slow and inconsistent, owing to poor government funding of family planning services. Additional to inadequate government funding of family planning intervention in Nigeria, there is a very wide gap between the estimated cost and the actual allocation of funds for procurement and distribution of family planning commodities [37]. For instance, between 2012 and 2016, the Federal government fulfilled only 11% of its FP2020 pledge to provide US$3 million annually for the procurement of family planning commodities [37].

According to the 2016 Appropriation Act, the government is referred to as provider of “counterpart” funding for family planning [37, 48]. This means that while donors serve as principal sources of funds, the government serves as a secondary funder of family planning intervention. However, with the ongoing withdrawal of donors and decline in donor contributions, there is a need for government to take on the role of principal funder of family planning in Nigeria [49, 50].

Although the Federal government prioritizes family planning interventions by making provisions in the annual budget and earmarking funds through special interventions (such as the SOML-PfR), the funding landscape for family planning interventions at the subnational level is dominated by external donations which are short-lived, unpredictable (in terms of amount and timing), and focused on a single area which is the procurement of commodities [51]. The nature of external funding influences subnational planning and effective implementation of family planning services. Program managers find it difficult to make or execute plans when they cannot rely on the amount of money or quantity of commodities that will be available. Moreover, the only guarantee that family planning commodities will reach the last mile of distribution is that State governments honor their commitments to funding the distribution and supply chain management system.

Currently, State governments feature minimally in the funding for family planning services, as service delivery is primarily driven by the commodities supplied by external donors and the fee-for-service payments that are made by clients [37]. The health budgets of many States in the country lump family planning intervention with reproductive health, and this increases the likelihood that family planning services will be overlooked in the budgetary allocations. The ongoing global advocacy for programme-based budgeting as a tool for increasing transparency, accountability and data-driven decision making, provides an opportunity for family planning to be categorized as a stand-alone programme.

Evidence from this review validates the need for the Federal and State governments, particularly, to step-up and take on a greater share of the responsibility for financing family planning intervention, including the procurement and supply of commodities, and service delivery. The advent of the Basic Health Care Provision Fund in Nigeria in which one percent of consolidated revenue fund is earmarked for provision of health services at the primary health care level provides an opportunity for further earmarking a percentage of this fund for family planning at the primary and local government levels. Domestic funding of FP can further be improved by earmarking at least one percent of the annual health budget to funding of FP programs.

However, this is a review article, and the findings may have been influenced by the following factors, (i) the personal viewpoints of the reviewers; (ii) the omission of relevant research due to literature search procedures; and (iii) errors in the translation of data from the primary source. Moreover, the estimates presented in this paper should be interpreted with caution since some of them are based on older available data.

In conclusion, Nigeria has made slow progress in achieving its family planning targets. Over a period of 28 years, total fertility rate declined by 0.7 children per woman, wanted fertility rate decreased by 1, and the gap between wanted fertility and actual fertility increased by 0.3. Over a five-year period, mCPR decreased by 0.6%, and unmet need for family planning increased by 2.5%. Nigeria still relies heavily on external donations for family planning intervention. This makes funding for family planning services to be unpredictable and imbalanced. This highlights the need for increased budgetary allocation and actual release of funds for FP interventions at national and subnational levels. Improving domestic resource contributions to family planning would contribute to improvements in service delivery, because more funds will be available to ensure procurement and uninterrupted supply of sufficient amounts of contraceptive commodities to the last mile. These findings are invaluable to policymakers and family planning program officers for advocating for more funding for family planning interventions**.** A detailed financial analysis is required to identify opportunities to leverage within the fiscal space to mobilize resources for family planning. Family planning programme managers will also require capacity building on how to use evidence to advocate for more domestic resources for family planning.

## Data Availability

The study dataset is available on request.

## Abbreviations

FP: Family Planning
mCPR: Modern Contraceptive Prevalence Rate
DRM: Domestic resource mobilization
UNFPA: United Nations Fund for Population Activities
SOML PforR: Saving One Million Lives Programme for Results
USAID: United States Agency for International Development
DFID: Department for International Development
SFH: Society for Family Health
